# Development of an IFNγ response‐related signature for predicting the survival of cutaneous melanoma

**DOI:** 10.1002/cam4.3438

**Published:** 2020-09-09

**Authors:** Baohui Hu, Qian Wei, Xueping Li, Mingyi Ju, Lin Wang, Chenyi Zhou, Lianze Chen, Zinan Li, Minjie Wei, Miao He, Lin Zhao

**Affiliations:** ^1^ Department of Pharmacology School of Pharmacy China Medical University Shenyang China; ^2^ Liaoning Key Laboratory of Molecular Targeted Anti‐Tumor Drug Development and Evaluation Liaoning Cancer immune peptide drug Engineering Technology Research Center Key Laboratory of Precision Diagnosis and Treatment of Gastrointestinal Tumors Ministry of Education China Medical University Shenyang China

**Keywords:** GEO, IFNγ response, prognosis, skin cutaneous melanoma, TCGA, tumor microenvironment

## Abstract

**Background:**

The tumor microenvironment (TME) plays a critical role in tumorigenesis, development, and therapeutic efficacy. Major advances have been achieved in the treatment of various cancers through immunotherapy. Nevertheless, only a minority of patients have positive responses to immunotherapy, which is partly due to conditions of the immunosuppressive microenvironment. Therefore, it is essential to identify prognostic biomarkers that reflect heterogeneous landscapes of the TME.

**Methods and materials:**

Based upon the ESTIMATE algorithm, we evaluated the infiltrating levels of immune and stromal components derived from patients afflicted by various types of cancer from The Cancer Genome Atlas database (TCGA). According to respective patient immune and stromal scores, we categorized cases into high‐ and low‐scoring subgroups for each cancer type to explore associations between TME and patient prognosis. Gene Set Enrichment Analyses (GSEA) were conducted and genes enriched in IFNγ response signaling pathway were selected to facilitate establishment of a risk model for predicting overall survival (OS). Furthermore, we investigated the associations between the prognostic signature and tumor immune infiltration landscape by using CIBERSORT algorithm and TIMER database.

**Results:**

Among the cancers assessed, the immune scores for skin cutaneous melanoma (SKCM) were the most significantly correlated with patients' survival time (*P* < .0001). We identified and validated a five‐IFNγ response‐related gene signature (UBE2L6, PARP14, IFIH1, IRF2, and GBP4), which was closely correlated with the prognosis for SKCM afflicted patients. Multivariate Cox regression analysis indicated that this risk model was an independent prognostic factor for SKCM. Tumor‐infiltrating lymphocytes and specific immune checkpoint molecules had notably differential levels of expression in high‐ compared to low‐risk samples.

**Conclusion:**

In this study, we established a novel five‐IFNγ response‐related gene signature that provided a better and increasingly comprehensive understanding of tumor immune landscape, and which demonstrated good performance in predicting outcomes for patients afflicted by SKCM.

## Graphical abstract

We established an effective five‐IFNγ response‐related gene‐based risk score, which has potential to be a novel prognostic signature and may provide insight into tumor immune microenvironment of cutaneous melanoma.

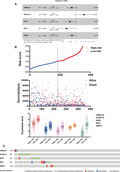

## 1 INTRODUCTION

Tumor microenvironment (TME) is the main location where tumor cells interact with the host immune system. Apart from tumor cells, the TME consists of varied heterogeneous components including immune cells, stromal cells, and extracellular components such as cytokines, chemokines, and hormones. The various components of the TME not only play important roles in tumor progression, immune escape, and metastasis, but also have a profound impact upon therapeutic efficacy of afflicted patients.^1^, ^2^, ^3^ For example, immunosuppressive cells within the TME play critical roles in promoting tumor immune escape and facilitate localized suppression of anti‐tumor immune responses by way of releasing immunosuppressive cytokines.^4^ Likewise, high levels of tumor infiltrating lymphocytes often correlate with a favorable survival in patients with skin cutaneous melanoma (SKCM) as well as other solid tumors.^5^ Emerging immunotherapeutic strategies involving immune checkpoint inhibitors (ICIs) have facilitated astounding improvements in the survival of patients afflicted by various types of cancers, however, the majority of afflicted patients have no stability in long‐term response to treatments, or continue to have relatively poor prognoses.^6^, ^7^, ^8^ The limited efficacy of these immunotherapies has been at least partly attributed to the immunosuppressive effects of TME. Even when considering just one type of relatively distinct cancer, the contexture and organization of immune infiltrates can be highly heterogeneous.^9^ Consequently, it is critical to understand the molecular composition and function of TME to facilitate effective diagnosis, prognosis, mitigations, and immunotherapeutic responsiveness of patients afflicted by cancers.

The Cancer Genome Atlas (TCGA) has helped to further cancer diagnostics and treatments by having provided comprehensive‐ and systematic‐based information for the genomics of varied cancers. Genomic and transcriptomic landscapes of tumors have been identified as key elements to be understood in order to define the dynamics of TME.^10^ Exploration of tumor immune response based upon gene expression profiles has elucidated significant roles played by tumor infiltrating immune cells.^11^

In our study, the main goal we sought to accomplish was to explore TME‐related prognostic biomarkers, which could also contribute to facilitating the identification of heterogeneous tumor immune landscapes and response effects of ICIs. Yoshihara et al described a novel algorithm called “Estimation of STromal and Immune cells in MAlignant Tumor tissues using Expression data” (ESTIMATE), which was designed to facilitate inference of proportions of immune and stromal components within the TME.^12^ Several studies have effectively applied ESTIMATE to explore microenvironments of breast cancer, glioma, and urothelial cancer.^13^, ^14^, ^15^ Thus, we used the ESTIMATE algorithm to calculate immune and stromal scores for distinct types of tumors which were all approved by FDA (Food and Drug Administration) for immunotherapy‐based treatments.^16^ The association of the scores and overall survival (OS) of these patients revealed that the immune scores of SKCM were most significantly correlated with patients' survival. As SKCM cells are highly immunogenic, we then focused upon the analysis of SKCM data. By using GSEA analysis, we found that the genes related to IFNγ response signaling pathway were markedly enriched in samples with high immune scores for respective patients. IFNγ affects tumor cell immunogenicity directly and is of critical significance in promoting tumor cell recognition and elimination.^17^ Tumor IFNγ expression has been identified to be closely correlated with favorable clinical outcome for multiple cancer types.^18^ Several IFNγ‐related gene profiles have been indicated to be critical markers of expected positive reactions to immune checkpoint blockade therapy.^19^ Based upon the findings from those assessments, we expected to shift our focus to exploring IFNγ response‐related prognostic signatures for patients with SKCM. Lastly, we sought to develop and assess a final five‐mRNA (UBE2L6, PARP14, IFIH1, IRF2, and GBP4) risk‐based model, which we hoped, could effectively predict clinical outcomes for patients as well as depict the tumor immune infiltration landscape of SKCM.

## 2 MATERIALS AND METHODS

### 2.1 Data collection and processing

Fragments per Kilobase Million (FPKM) expression profiles for patients afflicted with 11 types of tumors were obtained from the TCGA data portal (https://tcga‐data.nci.nih.gov/tcga/). Exclusion criteria included: (a) patients without complete clinical survival information, and (b) any duplicate samples. The “ESTIMATE” package (https://sourceforge.net/projects/estimateproject/) was applied to calculate immune and stromal scores for each patient. To validate the prognostic value of our risk model established from the training set, we downloaded the expression profiling of 210 SKCM afflicted patients and their respective complete prognostic information (GSE65904) based upon the GPL10558 platform from the Gene Expression Omnibus (GEO) (https://www.ncbi.nlm.nih.gov/geo/) database. Moreover, a cohort of 27 advanced melanoma patients receiving anti‐PD‐1 immunotherapy (GSE78220, GPL11154 platform) retrieved from GEO database was included in the current study to evaluate the immunotherapeutic value of the gene signature.

### 2.2 Survival analyses

Patients were included within groups for each cancer type, and then were divided into high‐ and low‐scoring groups in accordance with their median immune/stromal scores as the cutoff point. These groupings facilitated evaluation of associations between immune/stromal scores with patients' OS by using Kaplan‐Meier survival analyses and log‐rank tests.

### 2.3 Gene set enrichment analysis

GSEA (http://www.broadinstitute.org/gsea/index.jsp) analysis was conducted to elucidate enriched molecular mechanisms associated with long‐term survival of patients in the high immune score group based upon the MSigDB h.all.v6.2.symbols.gmt (Hallmarks) gene set collection.^20^ Gene sets with False Discovery Rate (FDR) < 0.25, and with normalized *P*‐values <.05 after the execution of 1000 permutations were considered as statistically significant.

### 2.4 Establishment of the prognostic gene signature

The significantly enriched in IFNγ response signaling pathway gene set was analyzed by using univariate and multivariate Cox hazard regression analyses to facilitate establishment of a prognostic risk model for SKCM afflicted patients based upon a risk predictive formula defined by the linear combination of model predictors weighted with the regression coefficient. In this formula, n indicates the number of selected genes, βi represents the coefficient of each gene from multivariate Cox regression analysis, and Xi displays the expression of each gene.

$$\text{Risk score}=\sum_{i=1}^{n}\beta i\times Xi$$

Patients were separated into high‐ and low‐risk subgroups based upon resultant median risk scores. We used the “survivalROC” package for time‐dependent ROC curve analyses to examine predictive accuracy of the risk model. In addition, we conducted Cox regression analyses and data stratification analyses to examine the predictive power of risk scores in patients afflicted by SKCM. Metascape (http://metascape.org/) was applied to facilitate functional annotations of pathways and biological functions of survival‐related genes.^21^

### 2.5 Assessing immune cells infiltration using CIBERSORT and TIMER database

CIBERSORT is a method that facilitates evaluation of abundances of cell types in complex tissues via a gene expression‐based approach.^22^ We used the CIBERSORT analytical approach and methods to extract information about the richness and proportions of 22 immune cell subtypes (including seven T cell types, naive and memory B cells, plasma cells, and NK cells) for both high‐, and low‐risk cohorts. To enhance deconvolution algorithm accuracy, only samples with *P*‐values <.05 were selected for further analysis.

The Tumor Immune Estimation Resource (TIMER https://cistrome.shinyapps.io/timer/) has been used to assess the abundance of immune cells for up to 32 types of cancers and respective data derived from TCGA.^23^ In our study, the correlations between the five‐gene signature expression and immune cells (including B cells, CD8+T cells, CD4+T cells, macrophages, neutrophils, and dendritic cells) in patients afflicted by SKCM were assessed by use of the TIMER database.

### 2.6 Statistical analysis

Immune scores and stromal scores were compared between different subgroups using One‐way analysis of variance (ANOVA) or unpaired t‐tests in GraphPad Prism 8 software. The levels of expression of mRNAs were log2 transformed prior to conducting Cox regression analyses and by using the R package “survival.” Kaplan‐Meier analyses and the log‐rank tests were applied to facilitate estimations of differences in OS between high‐ and low‐risk patients. We used χ^2^ (chi‐square) test to measure associations between our risk scores and clinical factors provided from information in the datasets. Genetic changes of the five‐gene signature were visualized by use of the cBioPortal for Cancer Genomics database (http://www.cbioportal.org/).^24^ The five mRNAs expression data of SKCM and normal tissues from TCGA and GTEx projects were analyzed in Gene expression profiling interactive analysis (GEPIA http://gepia.cancer‐pku.cn/) (*P* < .05 and |log 2 FC|>1).^25^ Distinctive infiltrations of the 22 immune cell types in the SKCM afflicted patient cohort were assessed using Wilcox tests. All statistical analyses in our study were performed using R version 3.5.3 (R software https://www.r‐project.org/), IBM SPSS 22.0 (IBM, Inc) and GraphPad Prism 8 (GraphPad Software Inc). *P* < .05 was indicated as the level of statistically significance.

## 3 RESULTS

### 3.1 Association of immune and stromal scores with 11 types of cancer prognosis

**FIGURE 1:**
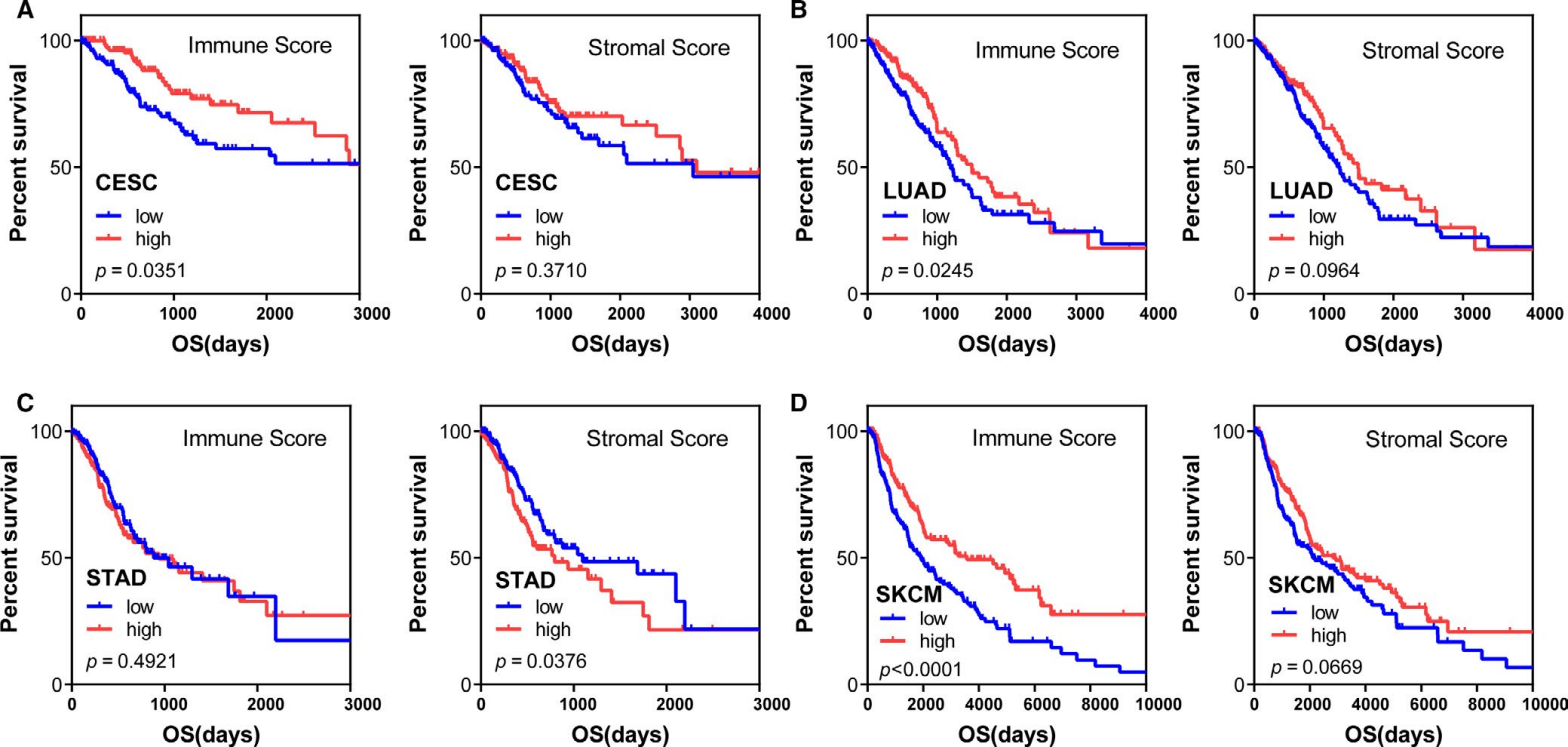
Kaplan‐Meier survival analyses of high vs low immune score and stromal score groups. A, cervical squamous cell carcinoma and endocervical adenocarcinoma (CESC). B, lung adenocarcinoma (LUAD). C, Stomach adenocarcinoma (STAD). D, Skin cutaneous melanoma (SKCM)

A workflow chart for our design and analysis phases is displayed in Figure S1. We downloaded protein‐coding gene expression profiles with corresponding survival information for patients afflicted by 11 types of cancers. Cancer afflicted cohorts enrolled in this study included patients afflicted by: bladder urothelial carcinoma (BLCA, 403), cervical squamous cell carcinoma, and endocervical adenocarcinoma (CESC, 291), head and neck squamous cell carcinoma (HNSC, 499), kidney renal clear cell carcinoma (KIRC, 525), kidney renal papillary cell carcinoma (KIRP, 283), liver hepatocellular carcinoma (LIHC, 365), lung adenocarcinoma (LUAD, 490), lung squamous cell carcinoma (LUSC, 488), prostate adenocarcinoma (PRAD, 495), stomach adenocarcinoma (STAD, 347), and skin cutaneous melanoma (SKCM, 454). According to the ESTIMATE algorithm, immune and stromal scores for each patient were calculated (Files [Link], [Link], [Link], [Link], [Link], [Link], [Link], [Link], [Link], [Link], [Link]). To detect potential correlation of OS with immune and with stromal scores, we divided patients into corresponding high‐ and low‐scoring subgroups within each cohort for each cancer type. Compared with other cancer types, Kaplan‐Meier survival analyses revealed that immune scores of patients afflicted by CESC (*P* = .0351), LUAD (*P* = .0245), and SKCM (*P* < .0001) were significantly associated with patients' survival time. Additionally, STAD afflicted patients with lower stromal scores had relatively better clinical outcomes than that of patients with higher stromal scores (*P* = .0376) (Figure 1A‐D). Specifically, we noticed that the immune scores of SKCM afflicted patients were most prominently associated with patients' prognosis. To validate the correlation of immune and stromal scores and SKCM afflicted patients' survival, we applied the ESTIAMTE algorithm to the GSE65904 cohort and got the same finding. We also found that patients with high immune scores had longer survival time than did patients in the low immune score group (Figure S2, *P* = .0125).

**FIGURE 2:**
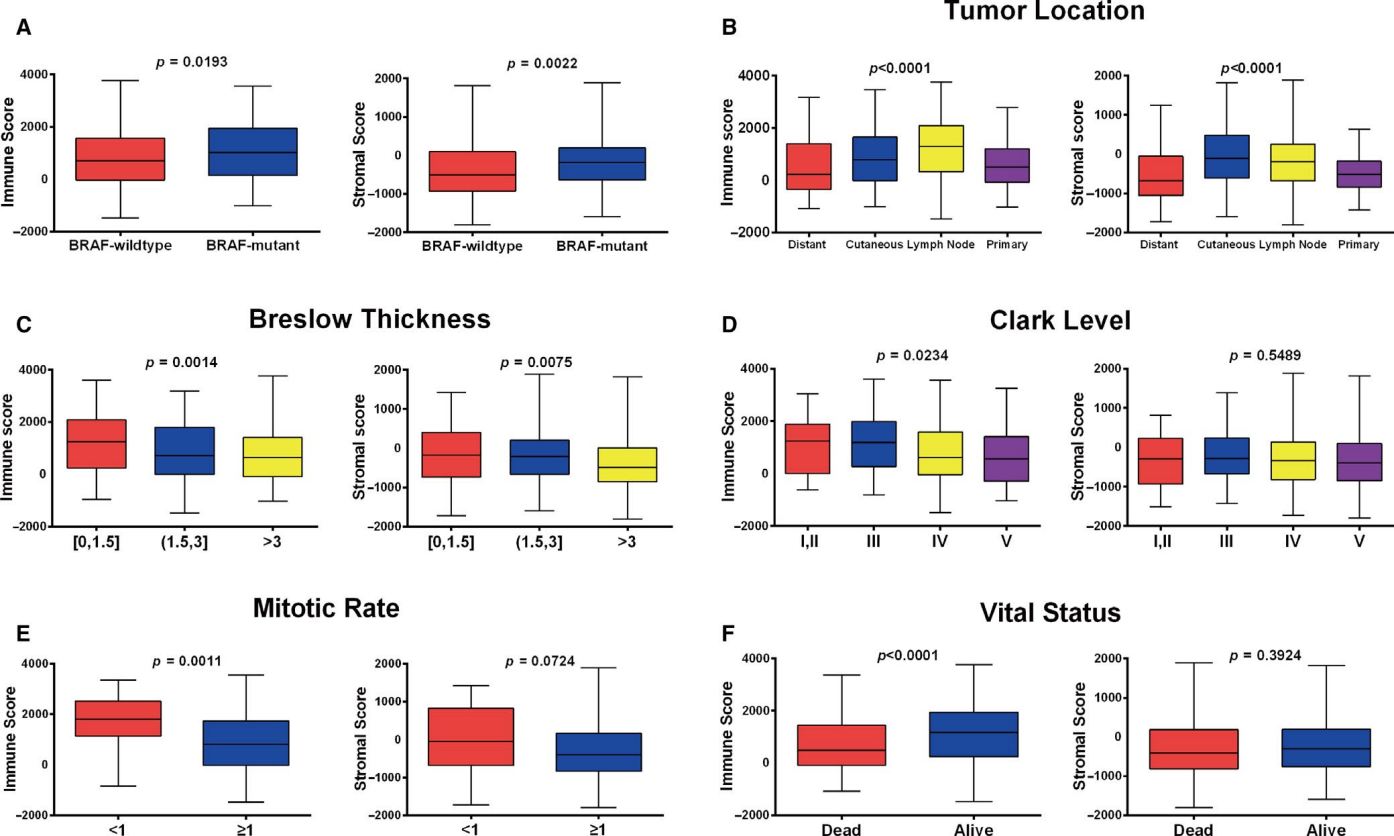
Immune scores and stromal scores are correlated with skin cutaneous melanoma (SKCM) clinicopathological features. A, Distribution of immune and stromal scores for BRAF wild‐type and BRAF mutant SKCM cases. Box‐plots indicate significant associations between BRAF mutation status and immune/stromal scores (*P* = .0193, *P* = .0022). B, Distribution of immune and stromal scores in different tumor locations. Box‐plots indicate significant associations between tumor locations and the level of immune and stromal scores (*P* < .0001, *P* < .0001). C, Distribution of immune and stromal scores of different Breslow thickness. Box‐plots indicate immune scores and stromal scores are both oppositely correlated with Breslow thickness (*P* = .0014, *P* = .0075). D, Distribution of immune and stromal scores of Clark levels. Immune scores display a negative correlation with Clark levels (*P* = .0234, *P* = .5489). E, Distribution of immune and stromal scores of patients with different mitotic rates. Box‐plots indicate immune scores of patients with mitotic rates <1 were higher than for mitotic rates ≥1 (*P* = .0011, *P* = .0724). F, Distribution of immune and stromal scores of patients with known vital status as alive or dead. Box‐plots indicate living patients had higher immune scores (*P* < .0001, *P* = .3924)

To assess measures of correlation between TME with SKCM, we plotted different score distributions following clinicopathological parameters of patients. SKCM is believed to be mostly driven by functionally based mutations of BRAF.^26^ In our assessments, we found that both immune and stromal scores were relatively higher in such types of BRAF mutants (Figure 2A). Moreover, average immune scores of regional lymph node cases ranked the highest of all four tumor locations, followed by scores representing regional cutaneous or subcutaneous tissue and primary tumor. The distant metastasis cases had the lowest representative immune scores. Similarly, the distributions of stromal scores were diverse and differed significantly with respect to different tumor locations (Figure 2B). As shown in Figure 2C‐E, immune scores were significantly correlated with important clinical features whereas stromal score distributions did not differ statistically for Clark levels and mitotic rates. Additionally, patients that survived and who remained alive had higher immune and stromal scores (Figure 2F). Other clinical parameters that significantly correlated with scores are listed in Table 1.

**TABLE 1:** The distribution of immune and stromal scores of skin cutaneous melanoma patients with different clinicopathological characteristics from The Cancer Genome Atlas database

Clinicopathological parameters	n	Immune Score			Stromal Score		
		Mean ± SEM	*t*	*P*‐value*	Mean ± SEM	*T*	*P*‐value*
Gender							
Male	282	879.8 ± 64.33	*t* = 2.042	**.0418**	−294.9 ± 41.82	*t* = 0.4950	.6208
Female	172	1099 ± 88.27			−262.1 ± 49.73		
Tumor location							
Cutaneous	73	902.4 ± 131.0	*F* = 13.57	**<.0001**	−72.09 ± 90.37	*F* = 11.27	**<.0001**
Lymph node	217	1277 ± 77.05			−176.5 ± 46.07		
Distant metastasis	64	528.2 ± 136.5			−551.8 ± 85.90		
Primary	97	597.4 ± 86.23			−497.0 ± 49.90		
Age							
<60	239	1037 ± 72.82	*t* = 1.506	.1328	−205.5 ± 46.14	*t* = 2.545	**.0113**
≥60	215	880.0 ± 74.85			−368.0 ± 43.58		
Pathologic stage (at original diagnosis)							
I	76	1256 ± 127.2	*F* = 6.751	**.0002**	−199.8 ± 81.67	*F* = 4.730	**.0030**
II	136	633.4 ± 78.56			−452.6 ± 52.96		
III	169	1076 ± 90.43			−231.9 ± 51.84		
IV	22	918.3 ± 228.8			−19.41 ± 173.8		
Breslow thickness (mm)							
≤1.5	104	1227 ± 111.5	*F* = 6.716	**.0014**	−167.6 ± 70.02	4.969	**.0075**
1.5‐3	77	850.7 ± 126.7			−233.5 ± 78.94		
>3	168	737.2 ± 79.59			−419.9 ± 50.06		
Ulceration							
Yes	163	736.5 ± 79.55	*t* = 2.619	**.0093**	−391.2 ± 48.60	*t* = 1.954	.0516
No	144	1064 ± 97.98			−239.3 ± 61.75		
Vital status							
Dead	207	717.9 ± 72.90	*t* = 4.374	**<.0001**	−312.4 ± 48.04	*t* = 0.8560	.3924
Alive	247	1168 ± 71.75			−257.3 ± 43.07		
Clark level							
I, II	23	1161 ± 211.8	*F* = 3.209	**.0234**	−339.8 ± 135.9	*F* = 0.7063	.5489
III	75	1185 ± 125.8			−207.0 ± 74.66		
IV	164	814.8 ± 88.10			−303.8 ± 55.63		
V	50	661.7 ± 149.8			−382.2 ± 101.5		
Mitotic rate							
<1	19	1794 ± 251.6	*t* = 3.325	**.0011**	21.39 ± 196.0	*t* = 1.808	.0724
≥1	149	884.0 ± 92.30			−300.9 ± 58.59		

* One‐way ANOVA analysis and unpaired *t* test were performed.

The *P*‐value showing statistical significance was marked with bold type.

### 3.2 Detecting genes enriched in IFNγ Response via GSEA

**FIGURE 3:**
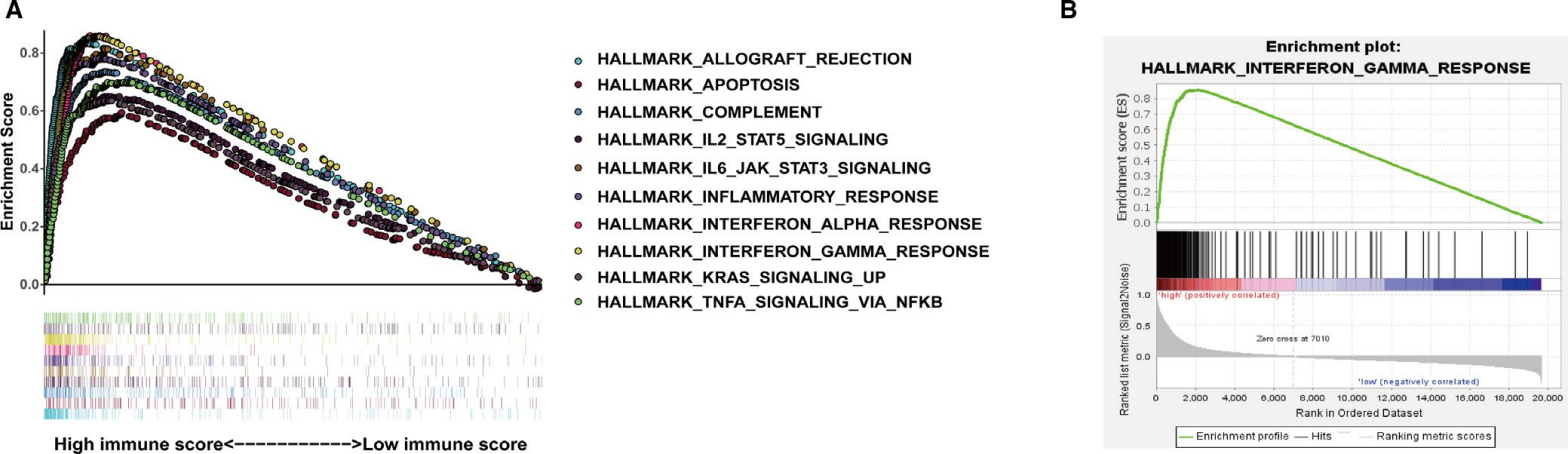
Enrichment plots of gene sets which were major differentiated between high and low immune score groups using gene set enrichment analyses. A, The top 10 enrichment gene sets in high immune score group. B, IFNγ response

We conducted GSEA to further investigate potential functional mechanisms leading to different prognosis for SKCM patients based upon stratification of their respective immune scores. In total, we identified 21 functions that were significantly enriched in the high immune score group (Table S1; File S12), which may have illustrated the underlying reasons for positive prognosis in the high immune score patient group (Figure 3A). Additionally, a total of 152 genes that displayed enrichment in the signaling pathway of IFNγ response were obtained for further analysis (Figure 3B; File S13).

### 3.3 Identification and construction of an IFNγ Response‐related gene signature associated with SKCM patients' survival

**FIGURE 4:**
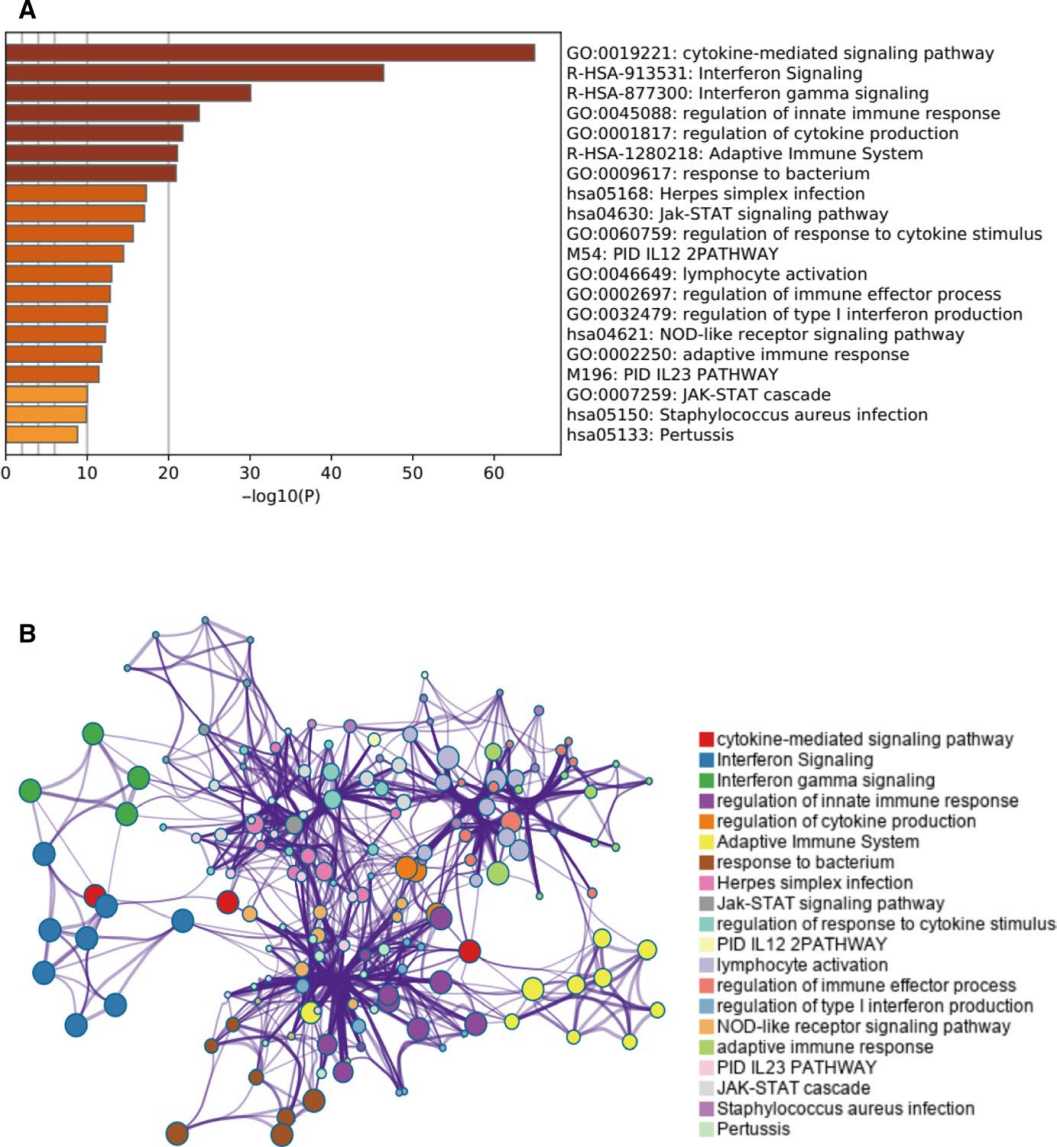
Metascape analysis. A, Enriched signaling pathways of the 116 prognostic‐value genes. B, The functional enrichment network

**FIGURE 5:**
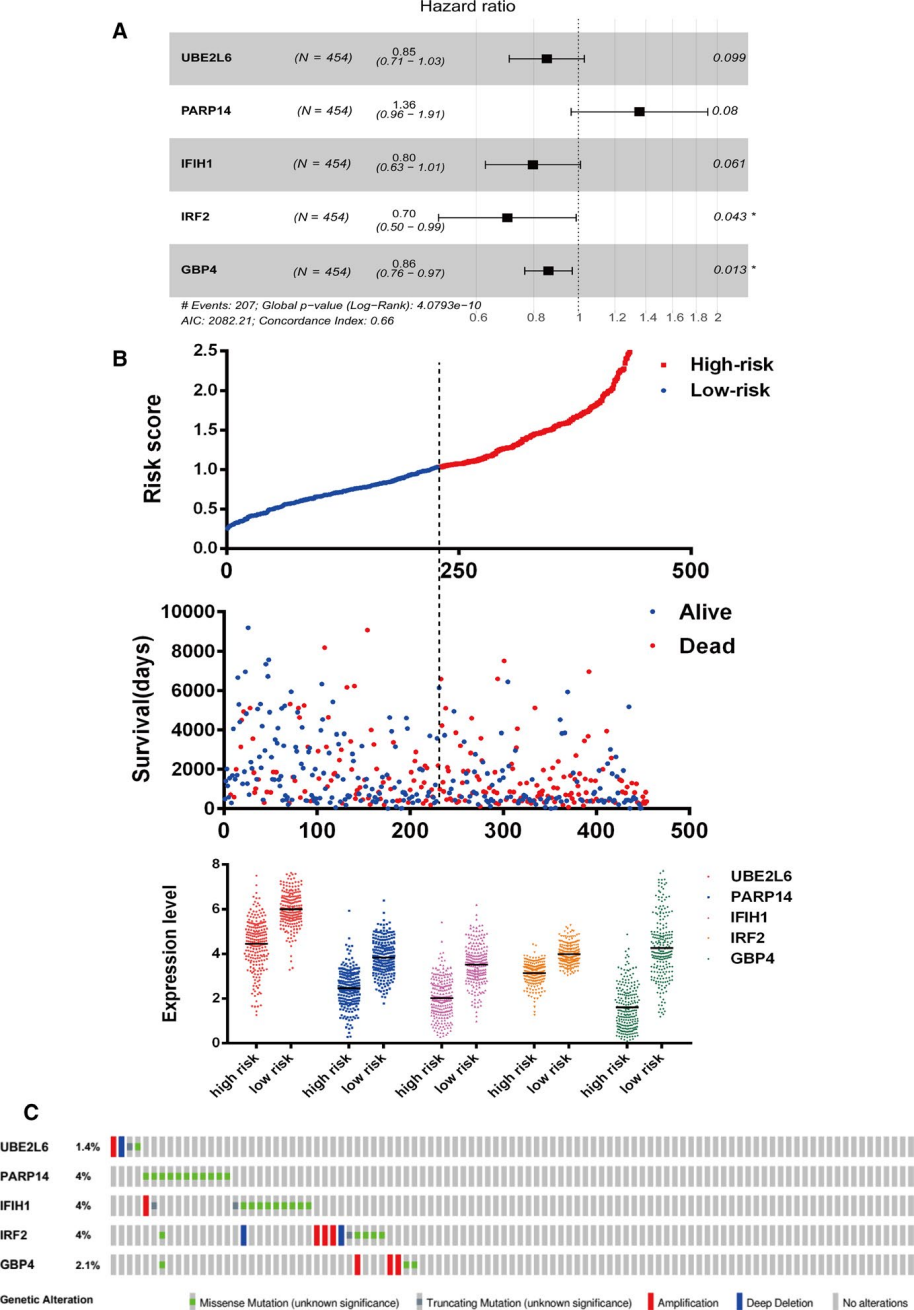
Distribution of risk score, patient survival time and expression of the five genes in the The Cancer Genome Atlas cohort. A, Five genes significantly correlated with overall survival derived from Cox regression analysis in skin cutaneous melanoma (SKCM) patients. B, Distribution of risk scores, patient survival time, status, and expression of five‐gene signature in high‐ and low‐risk groups. C, Alteration of the five genes in SKCM patients using the cBioportal database

To determine measures of association between the 152 genes and patient outcomes, we subjected these genes to univariate Cox hazard analysis. About 116 of the 152 genes were determined to have had significant prognostic value (*P* < .001, File S14). Metascape analyses indicated which were the top 20 clusters and enriched sets of the OS‐significant associated genes (Figure 4A,B). Among them, cytokine‐mediated signaling pathway, interferon signaling, and interferon gamma signaling were the most significantly enriched in the function of the OS‐associated genes. By ranking these genes in ascending order based upon respective *P*‐values, the top 15 predicted genes with the highest significance are listed in Table 2. To establish an optimal prognostic gene model, multivariate Cox regression was performed for and among the top 15 genes. A hazard ratio model consisting of five genes (UBE2L6, PARP14, IFIH1, IRF2, GBP4) was confirmed as the appropriate prognostic model for predicting OS of afflicted patients. Risk score = 0.30439 × expression of PARP14 −0.15824 × expression of UBE2L6 −0.22738 × expression of IFIH1 −0.35516 × expression of IRF2 −0.14989 × expression of GBP4 (Figure 5A). Subsequently, we ranked risk scores from low to high and divided sample data into low‐ and high‐risk groups according to median value and we found that all five genes were significantly upregulated in low‐risk patients (Figure 5B). Moreover, gene alteration in UBE2L6, PARP14, IFIH1, IRF2, and GBP4 were found to have occurred in only 1.4%, 4%, 4%, 4%, and 2.1% of sequenced cases respectively for data acquired from the OncoPrint schematic of cBioPortal (Figure 5C). By using GEPIA database, we found that the expression of all five genes was upregulated in patients afflicted by SKCM, however, differences for the levels of expression of IRF2 were not statistically significant (Figure S3).

**TABLE 2:** Univariate Cox analysis for survival‐predicted value of genes (top15)

Gene	HR	*z*	*P*‐value
GBP4	0.778934459	−6.136572874	8.43E‐10
IRF2	0.494163113	−5.939529589	2.86E‐09
UBE2L6	0.711962484	−5.927295314	3.08E‐09
NMI	0.624438806	−5.86769803	4.42E‐09
PARP12	0.665767813	−5.837071215	5.31E‐09
SAMD9L	0.706309057	−5.831177037	5.50E‐09
LAP3	0.650331556	−5.823474008	5.76E‐09
B2M	0.739388394	−5.62024323	1.91E‐08
SAMHD1	0.723394752	−5.595487658	2.20E‐08
IFIH1	0.703191011	−5.591126086	2.26E‐08
CXCL11	0.738148185	−5.576925346	2.45E‐08
CXCL10	0.837414034	−5.574184081	2.49E‐08
HLA‐DRB1	0.815715064	−5.543941084	2.96E‐08
DDX60	0.679572688	−5.531646212	3.17E‐08
PARP14	0.688482163	−5.503008717	3.73E‐08

Abbreviation: HR, hazard ratio.

**FIGURE 6:**
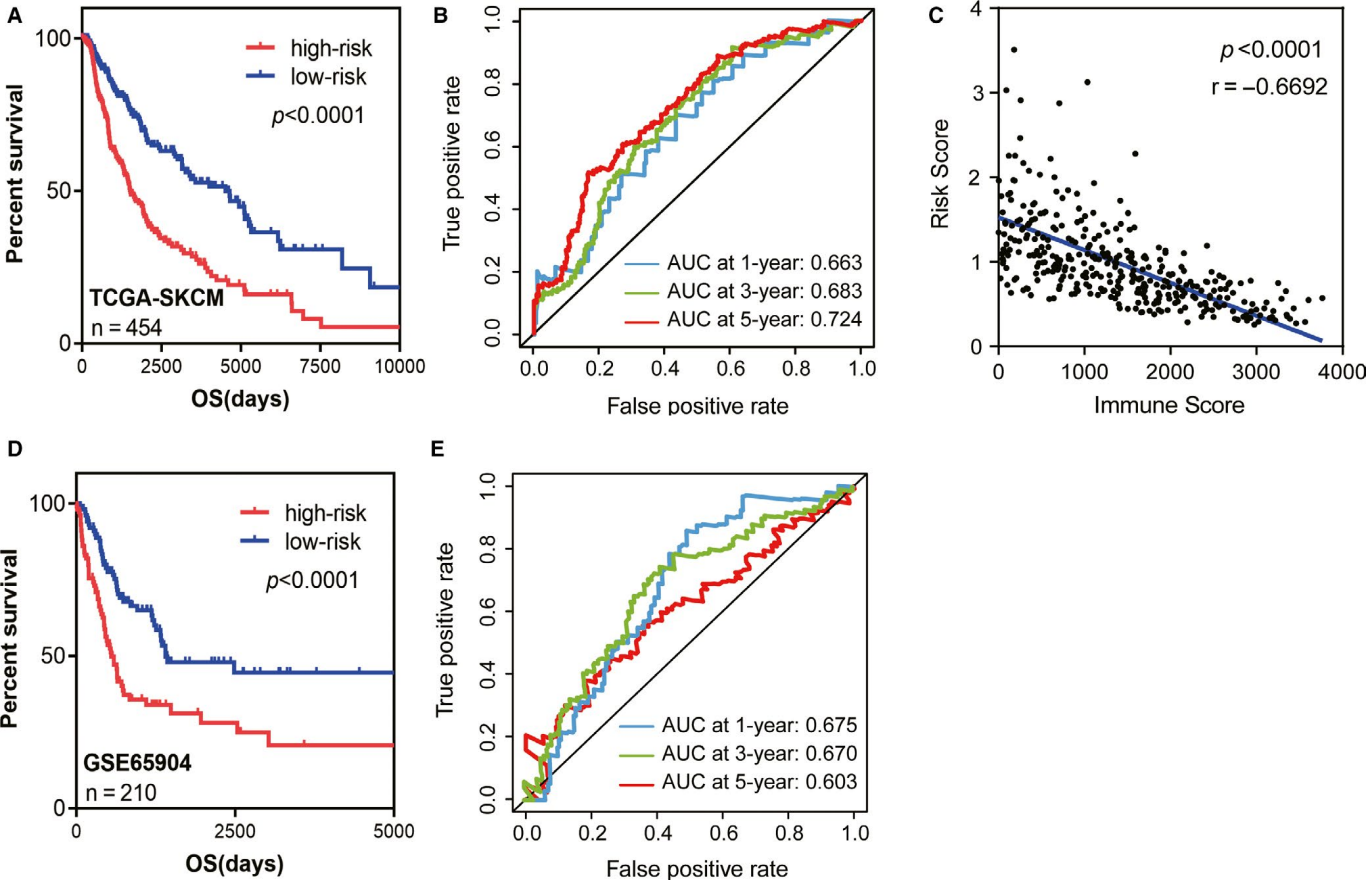
The five‐gene prognostic model predicted overall survival of patients with skin cutaneous melanoma (SKCM). A, Kaplan‐Meier survival curves for patients divided into high‐ and low‐risk groups in the TCGA‐SKCM cohort (n = 454, *P* < .0001). B, Time‐dependent receiver operating characteristic (ROC) plots displayed prediction efficacy of the five‐gene prognostic model in the SKCM patients from TCGA‐SKCM cohort at 1, 3, and 5 y. (C) The negative correlation of immune scores with risk scores in SKCM patients (*P* < .0001, *r* = −.6692). D, Kaplan‐Meier curves of the five‐gene signature in GSE65904 cohort (n = 210, *P* < .0001). E, Time‐dependent ROC plots displayed prediction efficacy of the five‐gene prognostic model in the SKCM patients from GSE65904 cohort at 1, 3 and 5 y

Kaplan‐Meier curve analyses indicated that the OS comparisons between the high‐ and low‐risk groups were notably different (median OS 1524 days vs 4507 days, *P* < .0001 Figure 6A). In addition, Pearson correlation analyses indicated that immune scores were negatively correlated with patient risk scores (*r* = −.6692, *P* < .0001, Figure 6C). Based upon established formulas, the test cohort (GSE65904 n = 210) was also stratified significantly by way of the five‐gene prognostic signature (median OS 550 days vs 1404 days, *P* < .0001, Figure 6D). The AUC (area under the curve) values of ROC (receiver operating characteristic) analysis at 5 years for the prognostic signature in TCGA cohort = 0.724 and for the GSE65904 cohort = 0.603, which suggested that the signature had satisfactory sensitivity and specificity with respect to predicting patients' survival (Figure 6B,E).

### 3.4 Evaluation of risk scores generated from the five‐mRNA signature as a prognostic indicator

After construction of a five‐gene prognostic model, we conducted Cox regression analyses to further evaluate effects of predicting prognosis independently. Univariate Cox regression analyses indicated that the IFNγ response‐related prognostic signature (HR = 2.247, 95% CI 1.698‐2.974, *P* < .001), pathological stage (HR = 1.640, 95% CI 1.210‐2.223, *P* = .001), AJCC‐T (HR = 1.992, 95% CI 1.421‐2.792, *P* < .001), AJCC‐N (HR = 1.773, 95% CI 1.307‐2.404, *P* < .001), Clark level (HR = 2.109, 95% CI 1.468‐3.029, *P* < .001), Breslow thickness (HR = 2.554, 95% CI 1.828‐3.569, *P* < .001), and ulceration status (HR = 1.983, 95% CI 1.402‐2.805, *P* < .001) were obviously associated with the survival of SKCM afflicted patients. After adjusting for other clinical parameters, the risk score (HR = 1.632, 95% CI 1.076‐2.475, *P* = .021), Breslow thickness (HR = 6.725, 95% CI 1.093‐41.362, *P* = .040), and ulceration status (HR = 1.493, 95% CI 1.005‐2.219, *P* = .047) were determined to be independent prognostic factors in multivariate analysis, which indicated that risk scores were remarkably correlated with OS of SKCM afflicted patients in the TCGA dataset (Table 3). We thus further investigated the association between the five‐gene signature and clinical characteristics. As shown in Table 4, the chi‐square test results suggested that the five‐gene based signature correlated with Breslow thickness (*P* < .001), ulceration status (*P* = .006), Clark level (*P* < .001), and AJCC‐T (*P* < .001).

**TABLE 3:** Univariate and multivariate Cox regression analysis for OS of five‐gene signature and other clinicopathological parameters

Parameters	Univariate COX		Multivariate COX	
	HR (95% CI)	*P*‐value	HR (95% CI)	*P‐*value
Risk score (high risk/low risk)	2.247 (1.698‐2.974)	**<.001**	1.632 (1.076‐2.475)	**.021**
Stage (advanced stage/early stage)	1.640 (1.210‐2.223)	**.001**	0.826 (0.249‐2.742)	.755
Gender (male/female)	1.171 (0.874‐1.570)	.290		
T (T3‐4/T1‐2)	1.992 (1.421‐2.792)	**<.001**	0.205 (0.033‐1.258)	.087
N (N1‐3/N0)	1.773 (1.307‐2.404)	**<.001**	2.756 (0.837‐9.075)	.095
M (M1/M0)	1.635 (0.834‐3.203)	.152		
Clark level (IV‐V/I‐III)	2.109 (1.468‐3.029)	**<.001**	1.253 (0.753‐2.087)	.385
Breslow thickness (≥2.0/<2.0 mm)	2.554 (1.828‐3.569)	**<.001**	6.725 (1.093‐41.362)	**.040**
Ulceration	1.983 (1.402‐2.805)	**<.001**	1.493 (1.005‐2.219)	**.047**

Abbreviation: HR, hazard ratio.

*Note:* The *P*‐value showing statistical significance was marked with bold type.

**TABLE 4:** The correlations between risk level and the characteristics of skin cutaneous melanoma patients

Parameters	N (high risk)	N (low risk)	Pearson χ^2^	*P* *
Gender				
Male	150	132	3.033	.082
Female	77	95		
Breslow thickness (mm)				
<2.0	50	76	14.058	**<.001**
≥2.0	135	88		
Ulceration				
Yes	99	64	7.475	**.006**
No	65	79		
Clark level				
Ⅰ‐Ⅲ	37	61	12.567	**<.001**
Ⅳ‐V	127	87		
Pathologic stage (at original diagnosis)				
Early (I and II)	117	95	2.290	.130
Advanced (III and IV)	91	100		
T				
T1‐2	45	72	13.647	**<.001**
T3‐4	140	96		
N				
N0	117	108	0.542	.462
N1‐3	85	91		
M				
M0	203	202	0.384	.535
M1	10	13		

* Pearson chi‐square test (χ^2^) analysis was performed.

The *P*‐value showing statistical significance was marked with bold type.

### 3.5 Independence of the five‐gene signature in the OS prediction from clinical characteristics

**FIGURE 7:**
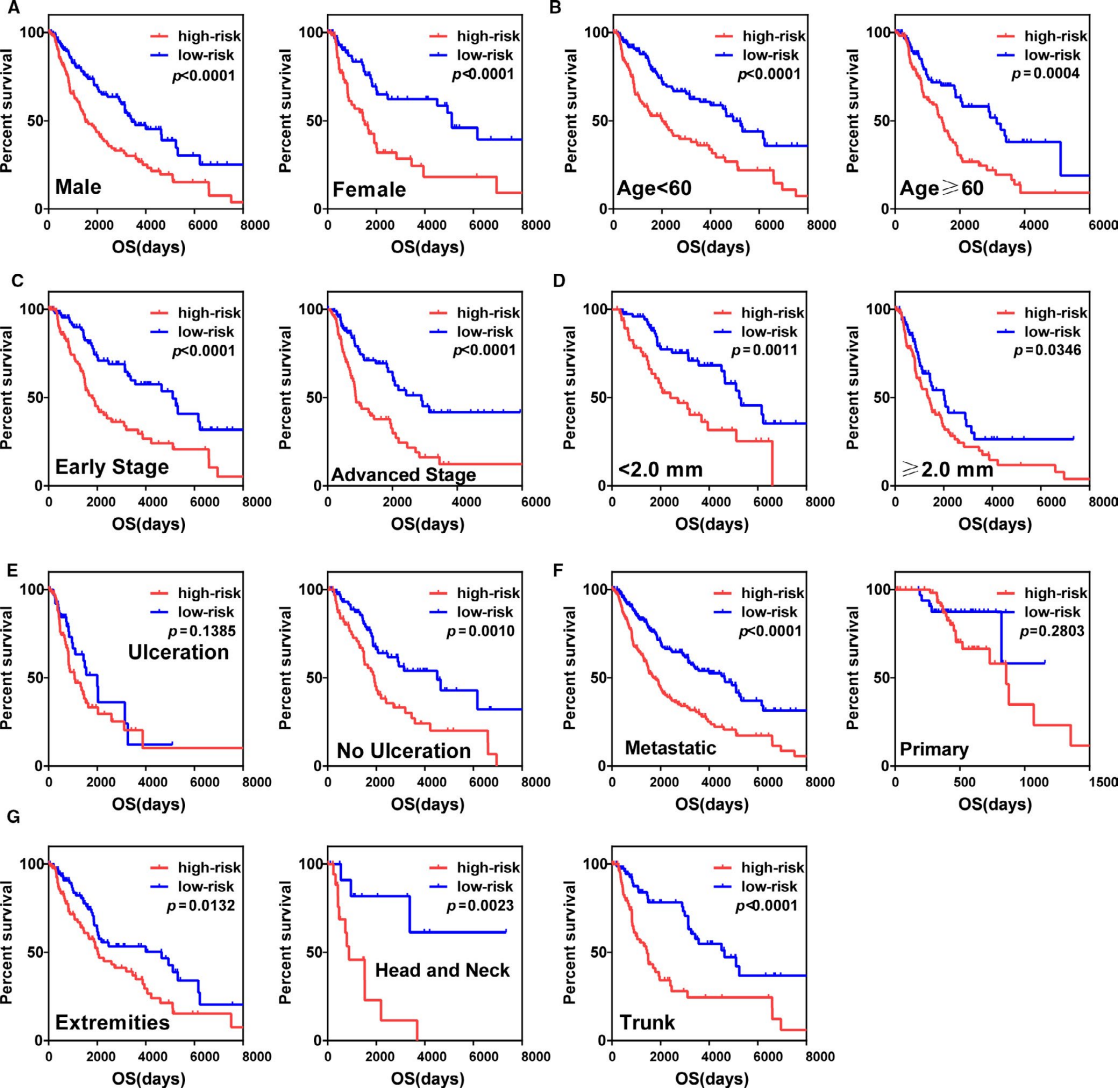
Kaplan‐Meier survival curves of patients in different clinical characteristics cohorts. Different clinical features including (A) male or female, (B) age < 60 or age ≥ 60 (C) early stage or advanced stage. (D) Breslow thickness < 2.0 mm or Breslow thickness ≥ 2.0 mm. (E) ulceration or no ulceration. (F) metastatic or primary tumor. (G) extremities, head and neck or trunk

Based upon the five‐gene signature, we used stratified analysis to further evaluate applicability and independence of risk scores. Kaplan‐Meier analyses indicated that independent of: gender, age, pathological stage (early stage or advanced stage), Breslow thickness (Breslow thickness <2.0 mm or ≥2.0 mm) and anatomic site (extremities, head and neck or trunk), the five‐gene signature had prognostic value for SKCM afflicted patients. However, for other clinical parameters such as ulceration status and tumor location, risk scores could not distinguish among the subgroups for ulceration or primary tumor effectively (Figure 7A‐G).

### 3.6 Estimating immunotherapeutic benefits and the immune infiltration landscapes in SKCM

To validate the ability of the prognostic signature to predict immunotherapeutic benefits, we assigned 27 patients treated with PD‐1 inhibitors in the GSE78220 cohort to high‐ and low‐risk subgroups. Although the difference was not statistically significant, patients with low‐risk scores were more likely to be immunotherapy responders than those in the high‐risk subgroup (one‐way ANOVA test, *P* = .1343; chi‐square test, *P* = .1083) (Figure S4A‐C). Moreover, we extracted patients with immunotherapy response information in TCGA‐SKCM cohort and found a survival benefit of low‐risk subgroup (*P* = .0277). The distribution of risk score in distinct response status to immunotherapy indicated that patients with stable disease had relatively higher risk scores than that of patients with complete response (one‐way ANOVA test, *P* = .1236; chi‐square test, *P* = .0986) (Figure S4D‐F).

**FIGURE 8:**
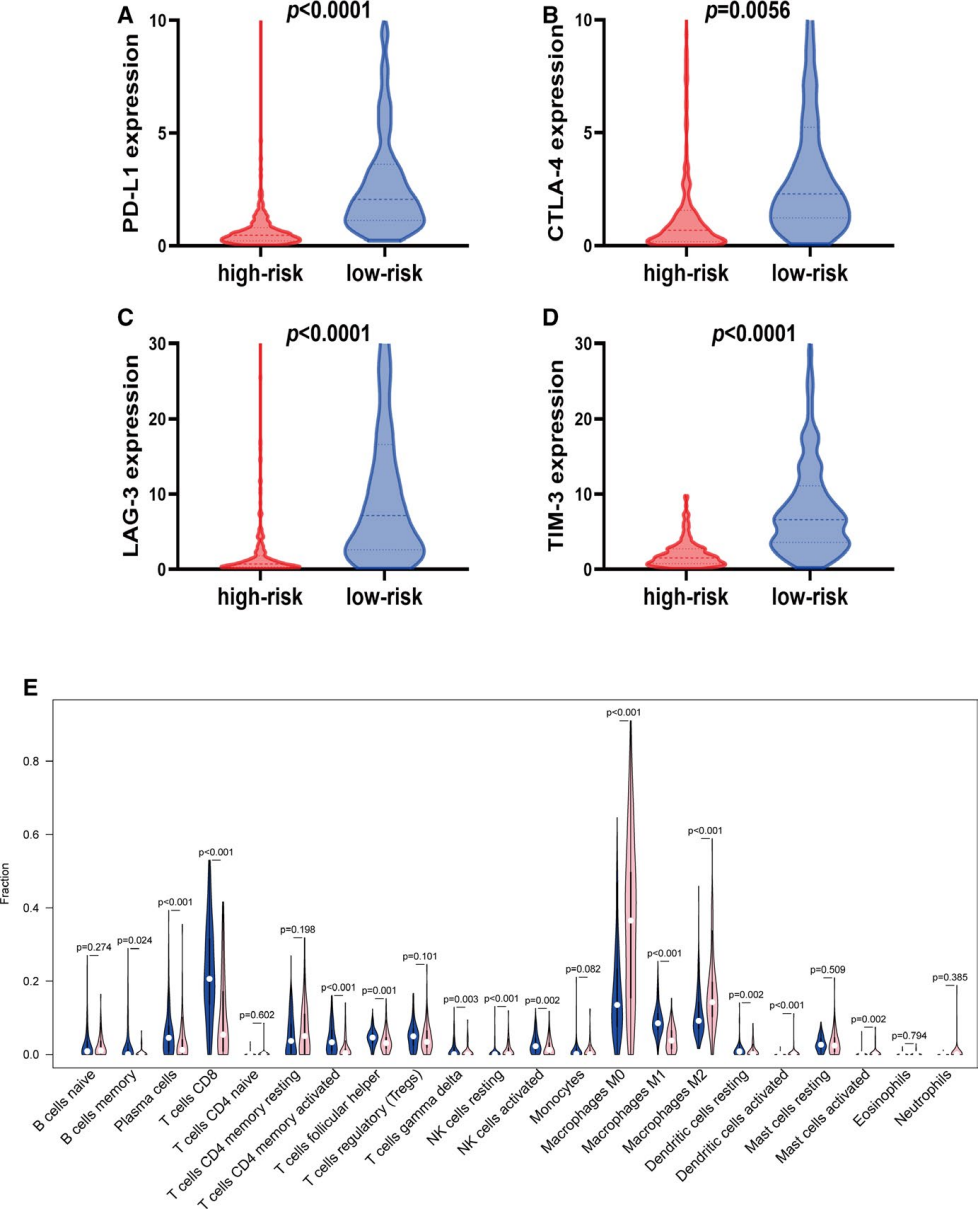
Relative proportions of immune cell expression in high‐ and low‐risk groups. A, Expression of PD‐L1 in low‐risk group was significantly higher than for high‐risk group (*P* < .0001). B, Expression of CTLA‐4 in low‐risk cohort was notably higher than for high‐risk cohort (*P* = .0056). C, Expression of LAG‐3 in low‐risk cohort was significantly higher than for high‐risk group (*P* < .0001). D, Expression of TIM‐3 in low‐risk cohort was relatively higher than for high‐risk group (*P* < .0001). E, The violin plot indicating relative proportions of immune cell expression distribution of TCGA‐SKCM patients stratified by the five‐gene signature into high‐ (pink) and low‐ (blue) risk groups

We then investigated the expression of immune checkpoint molecules involving programmed cell death 1 ligand 1 (PD‐L1), cytotoxic T‐lymphocyte‐associated protein 4 (CTLA4), lymphocyte activation gene‐3 (LAG‐3), and T cell immunoglobulin‐3 (TIM‐3) for patients that we stratified according to the five‐mRNA signature. Low‐risk patients had relatively higher expression of PD‐L1, CTLA‐4, LAG‐3, and TIM‐3 than was observed for patients in high‐risk cohorts (Figure 8A‐D).

CIBERSORT algorithm was applied to explore the proportions of 22 immune cell types in SKCM afflicted patients. Results revealed obviously distinct immune landscapes between patients in two subgroups. Patients in the low‐risk cohort (n = 166) had remarkably higher proportion of memory B cells, plasma cells, CD8 T cells, memory activated CD4 T cells, macrophages M1, follicular helper T cells, gamma delta T cells, activated NK cells, and resting dendritic cells compared to patients in the high‐risk cohort. In contrast, resting NK cells, macrophages M0, macrophages M2, activated mast cells, and activated dendritic cells were more present in the high‐risk sample cohort (n = 75) (Figure 8E).

**FIGURE 9:**
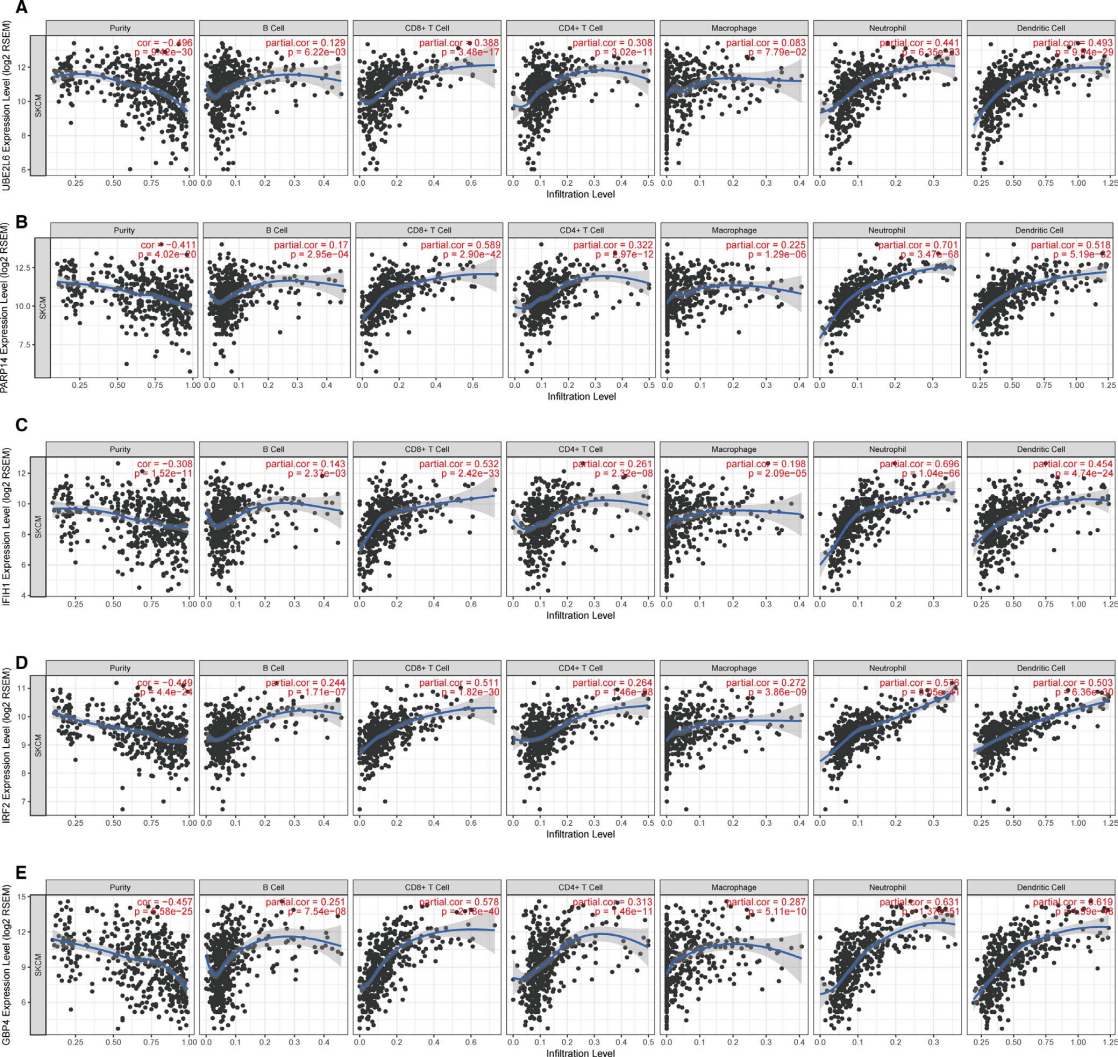
Correlations of expression of five genes with immune infiltration level in skin cutaneous melanoma. A, UBE2L6. B, PARP14. C, IFIH1. D, IRF2. E, GBP4

In addition, we performed correlation analyses to assess measures between the five genes and immune infiltration levels for SKCM afflicted patients by using the TIMER database. Scatter plots were generated indicated that the five genes expression levels were significantly negatively correlated with tumor purity (*P* < .05). Furthermore, PARP14, IFIH1, IRF2, and GBP4 expression showed remarkable positive association with infiltrating immune cells. UBE2L6 expression level also had significant association with infiltration levels of B cells, CD8+T cells, CD4+T cells, neutrophils, and dendritic cells, but did not for macrophages (Figure 9A‐E).

## 4 DISCUSSION

In recent years, increases in immunotherapy‐based research for cancer treatment have led to many advancements that have improved treatment outcomes for afflicted patients. However, effective immunotherapies, whether they are adoptive cell therapy or checkpoint blockade‐based approaches, have been limited for patients because of immunosuppressive barriers that exist in TME. The TME is known to be essential to the onset, development, progression, and relapse of many types of cancers.^27^ Thus, deeper explorations of the complexities within the TME may help to reveal novel biomarkers that could facilitate increased accuracy of prognosis for patients and their potential responsiveness to immunotherapy as well as improve therapeutic modulation. Therefore, we focused upon the TME to assess whether or not TME‐related genes contribute to OS in patients afflicted with malignant types of tumors.

Herein, we used the “ESTIMATE” approach to facilitate calculation of immune and stromal scores, which reflected levels of immune and stromal components infiltrating the TME for 11 types of cancer that have been approved for immunotherapy by FDA and respective data derived from the TCGA database. We found that immune scores were notably correlated with survival time of patients afflicted by CESC, LUAD, and SKCM, and stromal scores significantly correlated with OS in patients with STAD. These findings indicated that the TME was closely related to patient clinical outcome. Based upon the best knowledge available to us herein, the association between immune scores and SKCM was the most notable relationship of those tested, which is a finding consistent with the fact that SKCM is typically considered to be highly immunogenic.^28^ Consequently, we decided to assess tumor immune microenvironment‐related genetic factors of SKCM that may have contributed to OS in the TCGA database.

As an aggressive malignant neoplasia, SKCM has been estimated to cause about 55 000 annual deaths and accounts for approximately 232 100 (1.7%) cases of all newly diagnosed primary malignant tumors worldwide.^29^, ^30^ Accounting for more than 80% of skin cancer‐related mortalities, SKCM is one of the most fatal and treatment‐resistant carcinomas afflicting humans.^31^, ^32^ Patients with nonmetastatic SKCM have relatively high survival rates, whereas the 5‐year survival rate of sufferers with metastatic SKCM is only 14%.^33^ Encouragingly, immune checkpoint blockade antibodies such as pembrolizumab, nivolumab, and Ipilimumab targeting either PD‐1 or CTLA‐4 respectively have conspicuously altered the therapeutic landscape of SKCM in recent years.^34^, ^35^ However, the onset of and proclivity to the development of treatment‐based resistance and of recurrence of SKCM also persist. It has been estimated that approximately 60%‐80% patients receiving these treatments get no satisfying prognosis or lasting response.^36^, ^37^ Thus, SKCM is greatly suppressive and straightforwardly involved in immune evasion, which relies upon the interplay of SKCM cells with immune cells existing in the TME. In recent years, efforts to find effective prognostic biomarkers of SKCM have made some noteworthy progress. For instance, high levels of expression of SOX9, which is regulated by DNA methylation, was found to be correlated with decreased expression of tumor suppressor genes and was identified to be a negative prognostic factor in malignant SKCM.^38^ In addition, high baseline levels of plasma total cell‐free DNA (cfDNA) released from tumor cells also was a good predictor of poor prognosis for SKCM afflicted patients.^39^ Nevertheless, the levels of expression of a single gene could be affected by multiple factors or with consequently lower levels of sensitivity and specificity such as to be reliable prognostic biomarkers which could reflect tumor immune landscapes of SKCM, however, existent informative assessments are still lacking.

Instead of requiring definite differential gene thresholds, GSEA mainly concentrates upon assessing the levels of expression of annotated gene sets, which are coordinated differentially. Thus, we applied GSEA for 19 658 mRNAs from 227 SKCM afflicted patients with high immune scores and 227 SKCM patients with low immune scores. We found 21 functions that were significantly enriched in the high immune score group with nominal *P*‐values <.05. Then, we selected significantly related gene sets that were upregulated in response to IFNγ for further analyses. IFNγ is a major cytokine that facilitates both the adaptivity and innateness of immune systems. Additionally, effector T cell‐derived IFNγ is the most potent cytokine which contributes to high levels expression of PD‐L1 within the TME, indicating that upregulated responses to IFNγ signaling may be conducive in predicting positive responses to immune checkpoint inhibitor therapies.^40^ Previous research has reported that decreased levels of expression of specific genes from the interferon pathway correlated with poor prognosis for SKCM afflicted patients, however, an effective prognostic model associated with IFNγ response has not yet been established and applied in clinical setting.^41^ Thus, by applying Cox regression analyses in our research, we successfully identified a prognostic model with five IFNγ response‐related gene signatures for patients afflicted by SKCM. Considering that an ideal prognostic biomarker is one that can also stratify risk across independent cohorts effectively, we thus applied the GSE65904 cohort to further assess the practicality of our risk‐based model and found that the signature performed well in differentiating high‐ and low‐risk groups (*P* < .0001). SKCM has been described as a highly immunogenic cancer with heterogeneous histological and clinical features.^42^ Thus, we analyzed the independence and applicability of our five‐gene signature in samples obtained from different clinical characteristics. The results revealed that our signature was independent of gender, age, pathological stage, Breslow thickness, and anatomic site. As patients suffering from early stages of disease tended to have better potential for healing, our signature was identified to have had satisfactory predictive performance for patients with early‐staged diagnoses and Breslow thickness less than 2 mm. However, risk scores did not significantly predict outcomes for patients with ulcerations and primary locations. The reasons underlying these discrepancies should be further explored in appropriately designed follow‐up assessments. To validate the ability of the prognostic signature to predict immunotherapeutic benefits, we detected the distributions of risk scores in patients with distinct response status to immunotherapy in GSE78220 cohort and TCGA‐SKCM cohort. Although the difference was not statistically significance, the risk score was negatively associated with the immunotherapy response in the two cohorts and more immunotherapeutic responders appeared in the low‐risk groups than in the high‐risk groups. Additionally, we evaluated tumor infiltrating lymphocytes and predictive immune markers for immunotherapy of the signature and found that patients in low risk cohorts possessed higher measures of CD8 T cells, macrophage M1, as well as other immune cells related to infiltration, which indicated that this five‐gene signature might have potential as immunotherapy target thereby warranting further analyses.

Among the four protective genes, UBE2L6 gene, encoding the ISG15 (IFN‐stimulated gene 15)‐conjugating enzyme UbcH8, was found to be correlated with apoptosis of cervical cancer.^43^ Activation of IFIH1 (also known as MDA5) in tumors has been identified to facilitate re‐activation of tumor‐specific T cells which are otherwise functionally defective within the TME and to impede tumor‐induced immunosuppression in an IFN‐dependent manner.^44^, ^45^ The expression of GBP (Guanylate‐binding protein) has thus mostly been thought to be induced by IFNγ in various types of cells and is thought to help defend vertebrate cells against multiple invading pathogens and has antitumor activity.^46^ Additionally, GBP genes have been identified to be positive prognostic biomarkers for SKCM in afflicted patients.^47^ IRF2, as an IFN regulatory transcription factor has vital implications in approaches to assess cancer progression and for immunotherapy by way of both positively regulating the MHC pathway and down regulating PD‐L1 expression.^48^ Previous research has indicated that IRF2 is a crucial downstream target of oncogenic KRAS‐mediating immune suppression. What is more, enforced expression of IRF2 can inhibit an over resistance of tumors expressing KRAS with respect to PD‐1 based therapies.^49^ Poly (ADP‐ribose) polymerase (PARP)14 is a member of the PARP family of proteins and has been demonstrated to promote lymphomagenesis driven by persistent overexpression of the oncogene c‐Myc.^50^ Furthermore, PARP14 promoted pancreatic cancer cell proliferation, anti‐apoptosis, and GEM (gemcitabine) resistance via its relationship with the NF‐κB signaling pathway, which agrees our research that it has high potential to be an effective drug‐based target.^51^

The five‐gene signature, which we designed to identify the comprehensiveness of TME, is a biomarker with use for predicting prognosis and for guiding more effective immunotherapy‐based treatment strategies. Herein, we established a five‐gene signature to facilitate predictions of OS and to gain insight into the TME of SKCM‐based afflictions. The limitations of our study should also be acknowledged. Firstly, the most of our analyses and subjects are derived from data for Caucasians, and certain clinical features such as genetic factors and measures related to sun exposure are insufficient. Furthermore, as not all patients with low risk scores showed favorable response to immunotherapy, we should integrate more clinical parameters into the scoring system to ameliorate the prediction accuracy. Further studies should be directed such that biological functions of the five selected genes and validation of our risk score are assessed by way of actual clinical and laboratory‐based experiments.

## 5 CONCLUSION

In conclusion, our findings established an effective five‐IFNγ response‐related mRNA‐based risk score, which has the potential to be a novel prognostic signature and which may provide insight into tumor immune microenvironments of SKCM affliction. Further studies can be undertaken based upon our findings and should focus upon better understanding the function and molecular mechanisms underlying the genes we assessed and should seek to validate the effectiveness and applicability of our signature in clinically based experiments.

## CONFLICT OF INTEREST

The authors declare no conflict of interest.

## AUTHOR CONTRIBUTIONS

Conception, study design, and drafting the manuscript: Baohui Hu and Qian Wei. Acquisition and analysis of data: Baohui Hu, Qian Wei, Xueping Li, Mingyi Ju, Lin Wang. Interpretation of data and statistical processing: Chenyi Zhou, Lianze Chen, Zinan Li. Project administration and revision of the manuscript: Minjie Wei, Miao He, and Lin Zhao. All authors read and approved the final manuscript.

## Supporting information

Fig S1

Fig S2

Fig S3

Fig S4

Supplementary Material

Supplementary Material

Supplementary Material

Supplementary Material

Supplementary Material

Supplementary Material

Supplementary Material

Supplementary Material

Supplementary Material

Supplementary Material

Supplementary Material

Supplementary Material

Supplementary Material

Supplementary Material

## Data Availability

All datasets were adopted in this study are available in the TCGA (https://tcga‐data.nci.nih.gov/tcga/) and GEO (https://www.ncbi.nlm.nih.gov/geo/) database.
