# Supplementary figures and images for: Development of an IFNγ response‐related signature for predicting the survival of cutaneous melanoma
Source: Cancer Med. 2020 Sep 9;9(21):8186–201. doi: 10.1002/cam4.3438 (PMC7643661; doi:10.1002/cam4.3438)

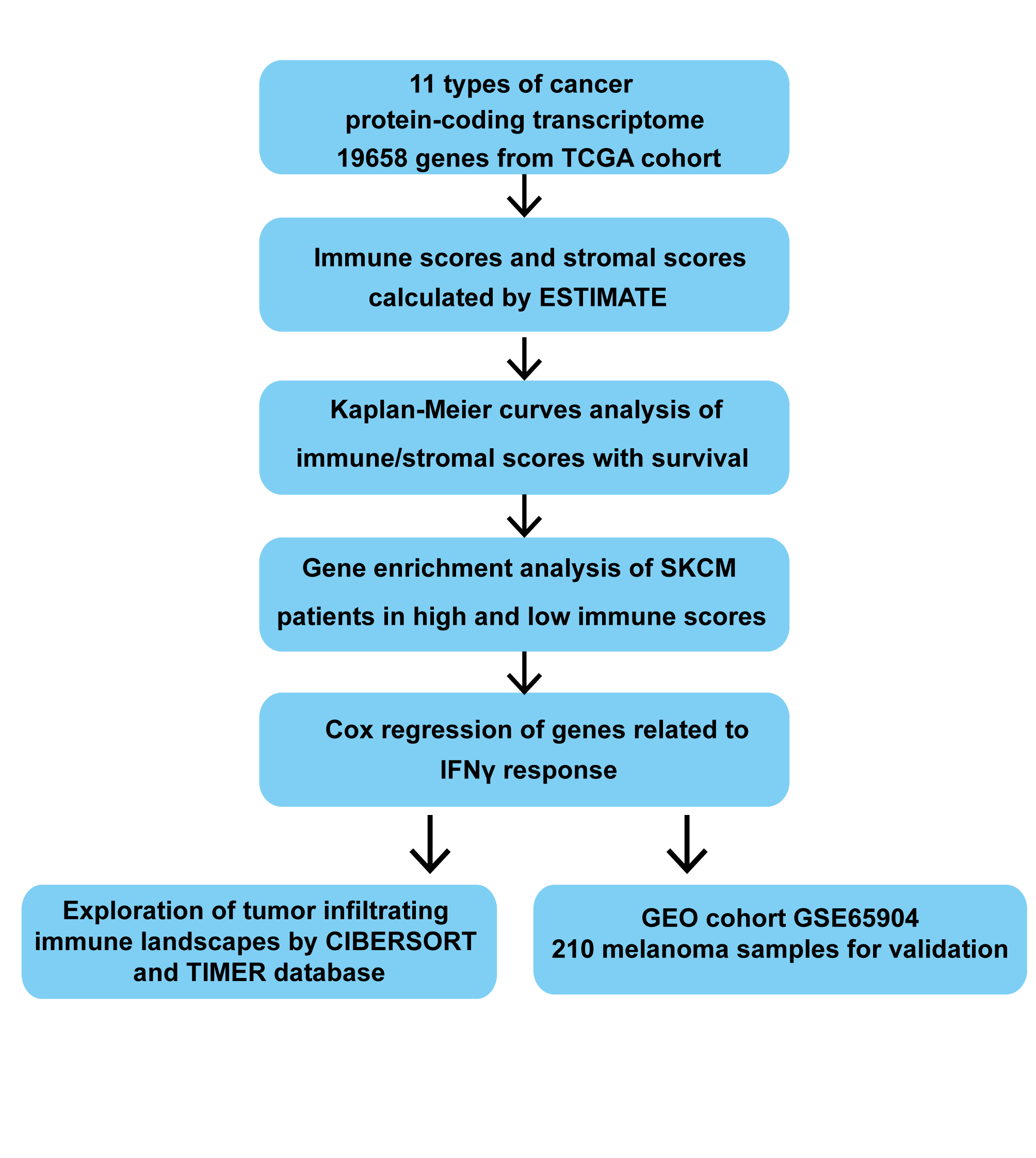

Supplement: Supplementary file 1 — Fig S1 [file CAM4-9-8186-s001.tif]

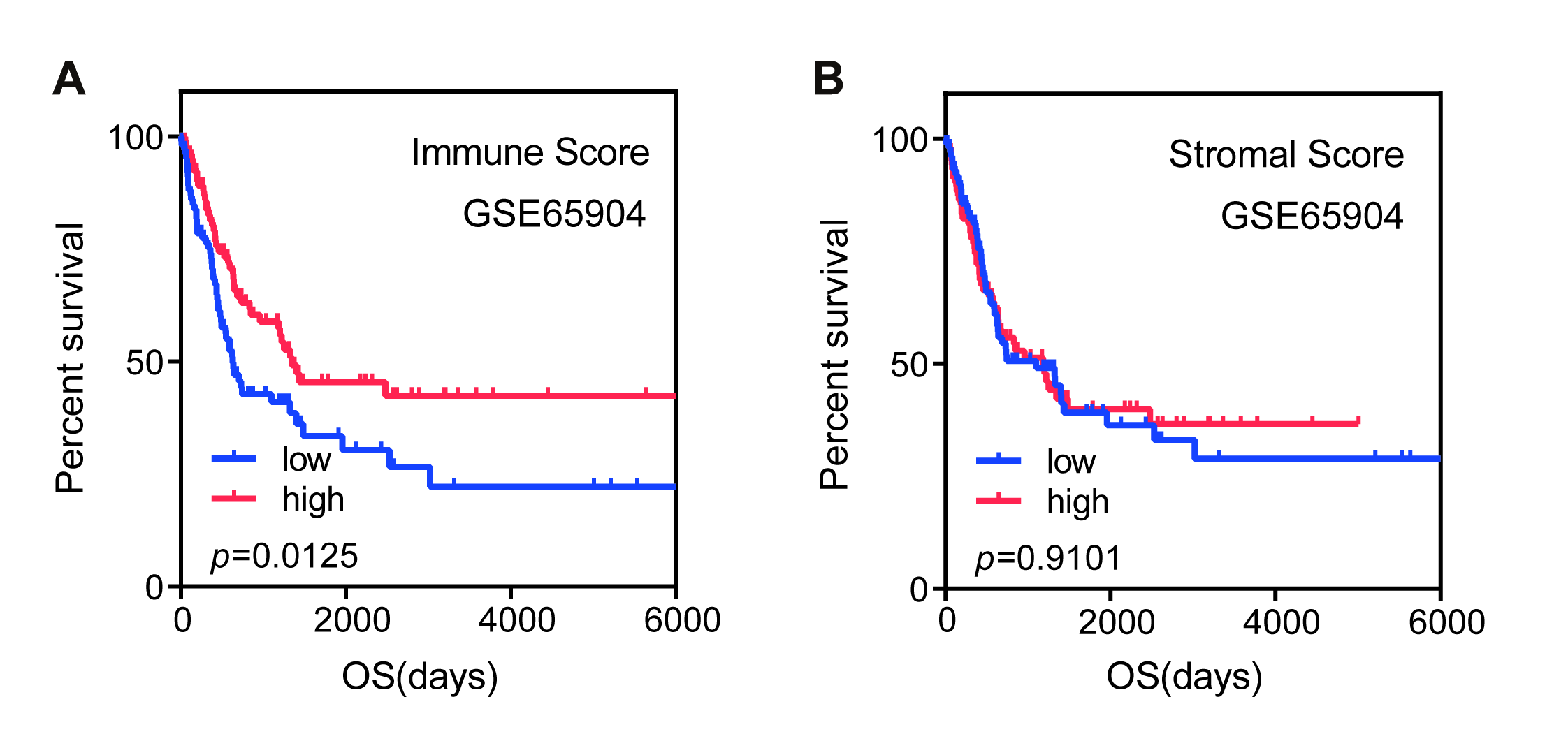

Supplement: Supplementary file 2 — Fig S2 [file CAM4-9-8186-s002.tif]

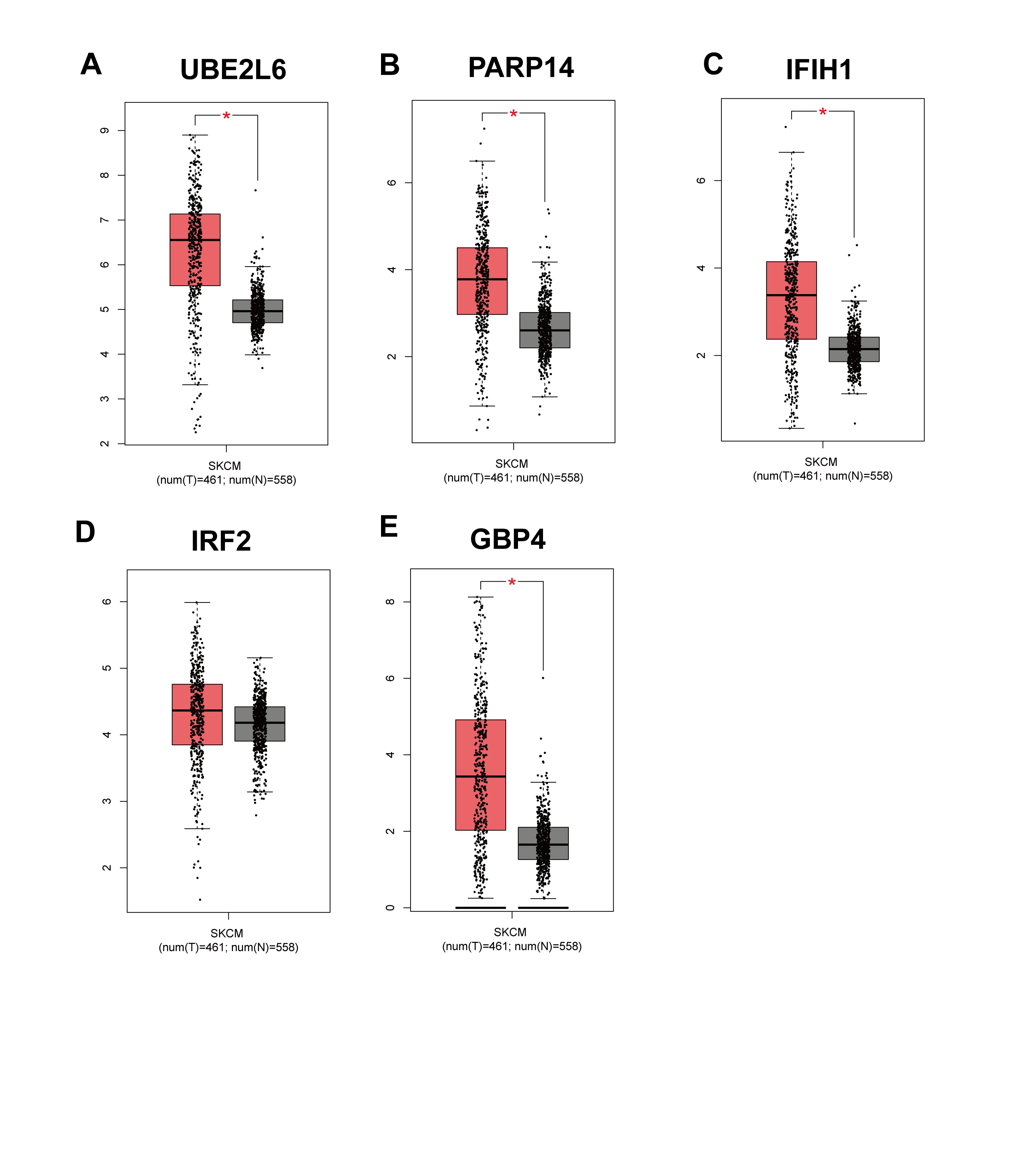

Supplement: Supplementary file 3 — Fig S3 [file CAM4-9-8186-s003.tif]

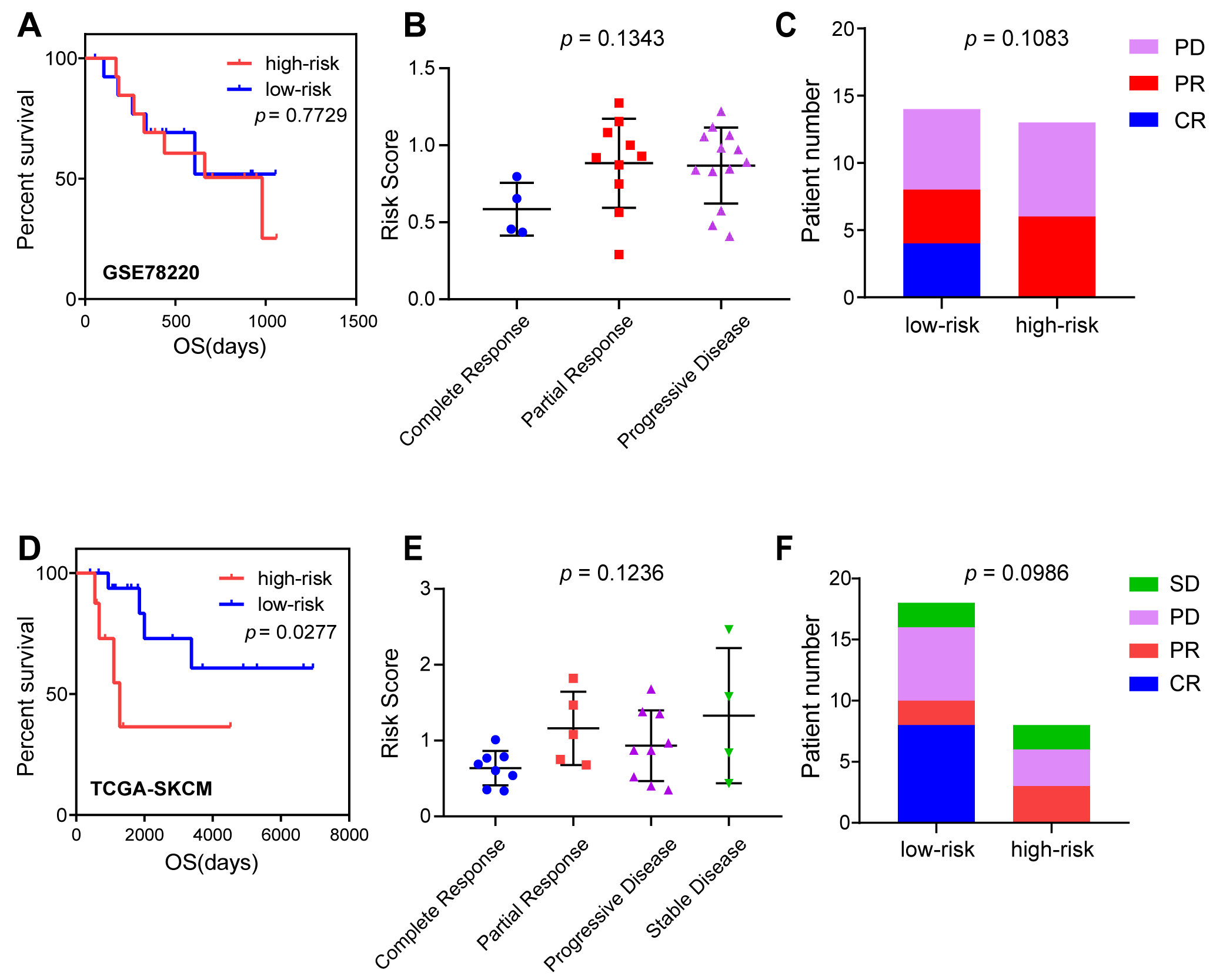

Supplement: Supplementary file 4 — Fig S4 [file CAM4-9-8186-s004.tif]
