# Supplementary material for: Development of an IFNγ response‐related signature for predicting the survival of cutaneous melanoma
Source: Cancer Med. 2020 Sep 9;9(21):8186–201. doi: 10.1002/cam4.3438 (PMC7643661; doi:10.1002/cam4.3438)
Supplement: Supplementary file 16 — Supplementary Material [file CAM4-9-8186-s016.docx]

| **Name** | **Size** | **ES** | **NES** | **NOM *p*** | **FDR *q*** | **FWER *p*** | **Rank at max** | **Leading edge** |
| --- | --- | --- | --- | --- | --- | --- | --- | --- |
| HALLMARK_COMPLEMENT | 199 | 0.734266 | 2.837983 | 0 | 0 | 0 | 2350 | tags=43%, list=12%, signal=49% |
| HALLMARK_INFLAMMATORY_RESPONSE | 200 | 0.780048 | 2.772646 | 0 | 0 | 0 | 2140 | tags=53%, list=11%, signal=59% |
| HALLMARK_IL2_STAT5_SIGNALING | 200 | 0.648824 | 2.718547 | 0 | 0 | 0 | 2473 | tags=37%, list=13%, signal=41% |
| HALLMARK_ALLOGRAFT_REJECTION | 200 | 0.832294 | 2.603661 | 0 | 0 | 0 | 1763 | tags=64%, list=9%, signal=69% |
| HALLMARK_IL6_JAK_STAT3_SIGNALING | 86 | 0.811652 | 2.599168 | 0 | 0 | 0 | 2378 | tags=66%, list=12%, signal=75 |
| HALLMARK_APOPTOSIS | 160 | 0.593186 | 2.591384 | 0 | 0 | 0 | 3013 | tags=37%, list=15%, signal=43% |
| HALLMARK_INTERFERON_GAMMA_RESPONSE | 198 | 0.854786 | 2.517713 | 0 | 0 | 0 | 2174 | tags=77%, list=11%, signal=85% |
| HALLMARK_TNFA_SIGNALING_VIA_NFKB | 198 | 0.696994 | 2.496395 | 0 | 0 | 0 | 2467 | tags=42%, list=13%, signal=48% |
| HALLMARK_KRAS_SIGNALING_UP | 200 | 0.63511 | 2.494221 | 0 | 0 | 0 | 2543 | tags=33%, list=13%, signal=38% |
| HALLMARK_INTERFERON_ALPHA_RESPONSE | 96 | 0.858516 | 2.234529 | 0 | 2.84E-04 | 0.002 | 1778 | tags=78%, list=9%, signal=85% |
| HALLMARK_PI3K_AKT_MTOR_SIGNALING | 105 | 0.486244 | 2.028402 | 0 | 0.003465285 | 0.019 | 2685 | tags=28%, list=14%, signal=32% |
| HALLMARK_COAGULATION | 138 | 0.537647 | 2.027859 | 0 | 0.003176511 | 0.019 | 2667 | tags=25%, list=14%, signal=29% |
| HALLMARK_REACTIVE_OXYGEN_SPECIES_PATHWAY | 49 | 0.558524 | 1.945194 | 0 | 0.007098774 | 0.042 | 2903 | tags=35%, list=15%, signal=41% |
| HALLMARK_APICAL_SURFACE | 44 | 0.523284 | 1.936238 | 0 | 0.007110649 | 0.045 | 2346 | tags=25%, list=12%, signal=28% |
| HALLMARK_APICAL_JUNCTION | 200 | 0.448727 | 1.913877 | 0 | 0.009247329 | 0.066 | 2295 | tags=22%, list=12%, signal=24% |
| HALLMARK_P53_PATHWAY | 199 | 0.398375 | 1.836129 | 0.002101 | 0.01849508 | 0.133 | 2656 | tags=22%, list=14%, signal=25% |
| HALLMARK_KRAS_SIGNALING_DN | 200 | 0.412385 | 1.741732 | 0 | 0.033268865 | 0.227 | 2748 | tags=20%, list=14%, signal=23% |
| HALLMARK_TGF_BETA_SIGNALING | 54 | 0.494652 | 1.690022 | 0.023483 | 0.043160923 | 0.284 | 3651 | tags=35%, list=19%, signal=43% |
| HALLMARK_XENOBIOTIC_METABOLISM | 198 | 0.400564 | 1.67649 | 0.002141 | 0.044808336 | 0.298 | 3097 | tags=25%, list=16%, signal=30% |
| HALLMARK_UV_RESPONSE_UP | 158 | 0.350893 | 1.60987 | 0.004032 | 0.06050912 | 0.398 | 2584 | tags=21%, list=13%, signal=24% |
| HALLMARK_HYPOXIA | 197 | 0.363453 | 1.582432 | 0.031311 | 0.06938851 | 0.449 | 3571 | tags=26%, list=18%, signal=32% |

**Table S1: HALLMARKER Annotation in high immune score group.**
